# Anais Brasileiros de Dermatologia: 2016-2020 Administration. Actions and acknowledgements^☆^

**DOI:** 10.1016/j.abd.2020.12.001

**Published:** 2021-02-16

**Authors:** Sinésio Talhari, Bernardo Gontijo, Everton Carlos Siviero do Vale, Silvio Alencar Marques

**Affiliations:** a Universidade Federal do Amazonas, Manaus, AM, Brazil; b Fundação Alfredo da Matta, Manaus, AM, Brazil; c Universidade Nilton Lins, Manaus, AM, Brazil; d Universidade Federal de Minas Gerais, Belo Horizonte, MG, Brazil; e Universidade Estadual Paulista, Botucatu, SP, Brazil

The first edition of Anais Brasileiros de Dermatologia (ABD) was published in 1925. Uninterruptedly, it has maintained its trajectory of scientific dissemination.^1^ Among other events, a leap in quality of the journal occurred with its indexation in PubMed in 2009. From that moment on, ABD has gained international visibility; in 2010, the first impact factor (IF) was released: 0.337. It was the beginning of another trajectory. Our IF has been progressively increasing; we reached 1.121 in 2020.^2^ We are ahead of some competing journals, such as Hautarzt, Annales de Dermatologie et de Venereologie, and Giornale Italiano di Dermatologia, among others. However, we are still far from JAMA Dermatology, JAAD, JID, and many others.

Always aiming to increase the quality of the journal, we sought innovation that stimulates our main vocation. Thus, the Tropical/Infectoparasitary Dermatology section was created. The other, more established areas of dermatology also deserved attention. Following the global trend, the sections “Research Letters” and “Case Letters” were created. Some sections were removed.

Seeking international recognition of ABD, the current editors and the Board of the Brazilian Society of Dermatology (SBD), during Dr. José Antônio Sanches’ administration (2017/2018), decided that the journal would be edited by an international company. After careful assessment, Elsevier was chosen. With this partnership, we gained access to Ahead of Print and Science Direct. This meant the immediate publication of articles, soon after being approved by the editors.^3^

A year ago, ABD reached the same level of visibility as the main international dermatological journals.^4^

These changes allowed the increase in IF and in the journal's visibility. Currently, the number of international articles submitted to ABD is greater than that of national articles.

However, to attract national and international research articles, further advancements are needed. Research related to doctoral theses, cutting-edge research, and other relevant investigations do not reach ABD. Rather, they are submitted to journals with higher scores. We were Qualis B3 and now we are Qualis B2.

For ABD to grow and reach new accomplishments, the participation of the national dermatological community is paramount. We are over 10,000 dermatologists and we deserve respect in the international dermatological community.

It is important to emphasize that everything that was accomplished during this period was only possible with the participation of many collaborators and the support of the executive boards.

We thank the members of the SBD Deliberative Council who, in 2015, entrusted us with the direction of the ABD for the 2016-2020 period.

Since the beginning of our activities in 2016, we have had the unconditional support of all SBD departments. On behalf of Presidents José Sanches and Sérgio Palma, we would like to thank all colleagues on these boards.

In this task, the collaboration of countless reviewers of articles already published or awaiting publication was essential. It is impossible to name all of them. They have been and will be fundamental pieces for the credibility of ABD.

We would like to thank our operational base: Priscila Rudge, Vanessa Zampier, Nazareno Souza, Bruno Souza, Laryssa Novato. We also would like to thank all employees at SBD headquarters.

## Financial support

None declared.

## Authors’ contributions

Sinésio Talhari: Elaboration and writing of the manuscript; critical review of the manuscript.

Bernardo Gontijo: Elaboration and writing of the manuscript; critical review of the manuscript.

Everton Carlos Siviero do Vale: Elaboration and writing of the manuscript; critical review of the manuscript.

Silvio Alencar Marques: Elaboration and writing of the manuscript; critical review of the manuscript.

## Conflicts of interest

None declared.
